# Current status of bacteriophage therapy for severe bacterial infections

**DOI:** 10.1186/s40560-024-00759-7

**Published:** 2024-11-01

**Authors:** Teiji Sawa, Kiyoshi Moriyama, Mao Kinoshita

**Affiliations:** 1 University Hospital, Kyoto Prefectural University of Medicine, 465 Kajiicho, Kawaramachi-Hirokoji-Agaru, Kamigyo, Kyoto, 602-8566 Japan; 2 Department of Anesthesiology, School of Medicine, Kyorin University, Mitaka, Japan; 3 Department of Anesthesiology, Kyoto Prefectural University of Medicine, Kyoto, Japan

## Abstract

The increase in the incidence of antibiotic-resistant bacteria poses a global public health threat. According to a 2019 WHO report, approximately 1.27 million deaths were attributed to antibiotic-resistant bacteria, with many cases linked to specific bacterial species, such as drug-resistant *Pseudomonas aeruginosa* and *Staphylococcus aureus.* By 2050, the number of deaths caused by these bacteria is predicted to surpass that caused by cancer. In response to this serious situation, phage therapy, an alternative to antibiotic treatment, has gained attention. Phage therapy involves the use of viruses that target specific bacteria to treat infections. This method has proven effective in multiple clinical cases, particularly for patients with severe infections caused by multidrug-resistant bacteria. For example, there are reports of patients with systemic infections caused by multidrug-resistant *Acinetobacter* who recovered following phage administration and patients infected with panresistant *Pseudomonas aeruginosa* who were cured by phage therapy. A key feature of phage therapy is its high specificity. Phages infect only specific bacteria and eliminate them. However, this specificity can also be a disadvantage, as careful selection of the appropriate phage for the target bacteria is needed. Additionally, bacteria can develop resistance to phages, potentially reducing treatment effectiveness over time. Efforts are underway to select, combine, and improve phages to address these challenges. In Belgium, a national phage bank has been established, and in the United States, the University of California, San Diego, has founded Innovative Phage Applications and Therapeutics (IPATH), marking significant progress toward the clinical application of phage therapy in the country. As a result, phage therapy is emerging as a component of personalized medicine, offering a new treatment option against antibiotic-resistant bacteria. The clinical application of phage therapy is particularly important in life-saving treatments for patients with severe bacterial infections, and its use in conjunction with antibiotics could enhance therapeutic outcomes. Continued research and development of this therapy could provide hope for many more patients in the future.

## Introduction

The spread of antibiotic-resistant bacteria has posed a serious threat to global public health. According to a 2019 WHO report, antimicrobial resistance is responsible for 1.27 million deaths worldwide, with approximately 80% of these deaths linked to six bacterial species, including *Staphylococcus aureus* and *Pseudomonas aeruginosa* [1, 2]. If current trends persist, by 2050, the number of deaths caused by drug-resistant bacteria could surpass that caused by cancer, exceeding 10 million annually [3]. The most recent report, published by a team of international researchers in September 2024, estimates that over the next 25 years, the number of deaths caused by drug-resistant bacteria, which are unaffected by antibiotics, will exceed 39 million worldwide, with an additional 169 million related deaths [4]. In this alarming context, phage therapy, which is an alternative to traditional antibiotics, is gaining attention.

In 2016, a landmark case in which a patient suffering from a systemic infection caused by multidrug-resistant *Acinetobacter* fully recovered after receiving systemic phage therapy was reported [5]. Since then, a growing number of success stories have emerged, such as the treatment of a 15-year-old cystic fibrosis patient with disseminated *Mycobacterium abscessus* infection who received a triple-phage cocktail following a double lung transplant [6]. Another notable case involved a 30-year-old woman with a panresistant *Klebsiella pneumoniae* infection related to a fracture, in which a six-day phage therapy succeeded after nearly two years of failed antibiotic treatments [7]. Most recently, a young child infected with extensively drug-resistant *P. aeruginosa* following a liver transplant underwent 86 days of combination therapy with phages and antibiotics, leading to a full recovery without side effects and successful liver retransplantation [8].

Phage therapy is no longer a futuristic medical concept but rather a real, life-saving intervention for patients suffering from severe bacterial infections. In intensive care units, where managing intractable bacterial infections is at the forefront of life-saving care, phage therapy is emerging as a crucial tool. As its use has gained momentum in North America and Europe, clinicians are increasingly confronted with a critical choice: “Do we surrender and accept defeat from resistant infections, or do we take a chance on phage therapy?”. By reviewing recent case reports and the status of ongoing clinical trials, we sought to evaluate the current state of phage therapy.

## Characteristics of phage therapy

Phage therapy has various characteristics that differ from those of traditional drug-based treatments. These are succinctly summarized below (Table 1). Phages can be categorized into two types: lytic and lysogenic (temperate) [9–12]. Lytic phages infect host cells, causing them to burst and effectively killing the bacteria. In contrast, lysogenic phages integrate their genome into the host bacterium's DNA without immediately killing the bacteria. While lysogenic phages can eventually cause cell lysis, they rarely halt bacterial infections immediately and may inadvertently facilitate the spread of drug resistance or toxin genes among bacteria. For this reason, lytic phages are the preferred choice in phage therapy.

**Table 1:** Major characteristics of phage therapy

Characteristics	
Narrow specificity	• Phages target specific bacterial strains rather than broad ranges of species like antibiotics • Types of phages: Lytic phages: they kill bacteria by causing cell lysis and are commonly used in therapy Lysogenic phages: they integrate into bacterial DNA, sometimes spreading resistance genes • Phage selection: effective treatment requires selecting phages tailored to the bacterial strain
Bacterial resistance	• Mechanisms include surface receptor mutations, CRISPR–Cas, restriction enzymes, and abortive infection • Phage cocktails are used to prevent resistance and target multiple strains
Immunogenicity	• Phage proteins can trigger immune responses, reducing effectiveness in chronic infections • Neutralizing antibodies (IgM, IgG) can diminish treatment efficacy • Phage lysis can result in the release of bacterial toxins, causing inflammation, with adjuvant therapies being explored to reduce these effects
Inflammation caused by phage-induced bacterial lysis	• Phage-induced bacterial lysis can result in the release of bacterial components, causing acute inflammatory response

### Phages have a narrow range of effectiveness for bacteria

Phages typically exhibit high host specificity, targeting only specific strains within a bacterial species. Unlike antibiotics, which can act on a wide range of bacteria, natural phages do not have a broad-spectrum effect. Therefore, selecting effective phages tailored to the specific bacterial strains involved in infection is essential when applying phage therapy [13]. This approach often requires creating a phage library for each infection-causing bacterial strain or, in other words, for each patient. From this library, phages that show lytic activity against the targeted strains are selected through a screening process. As a result, incorporating personalized treatment plans to identify and select the appropriate phage is a critical aspect of effective phage therapy. To compensate for the narrow host range of phages, innovative research facilities maintain a library of phages that target specific bacterial species such as *Achromobacter xylosoxidans*, *Enterobacter cloacae*, *Enterococcus faecalis*, *Enterococcus faecium*, *Klebsiella aerogenes*, *Klebsiella pneumoniae*, *Staphylococcus aureus*, and *Staphylococcus pseudintermedius* [14]. They have established systems to rapidly and efficiently screen for adaptive phages, and by preparing cocktail phages that combine multiple phages, they can extend the scope of action against target bacteria.

### Bacteria acquire phage resistance

**Fig. 1:**
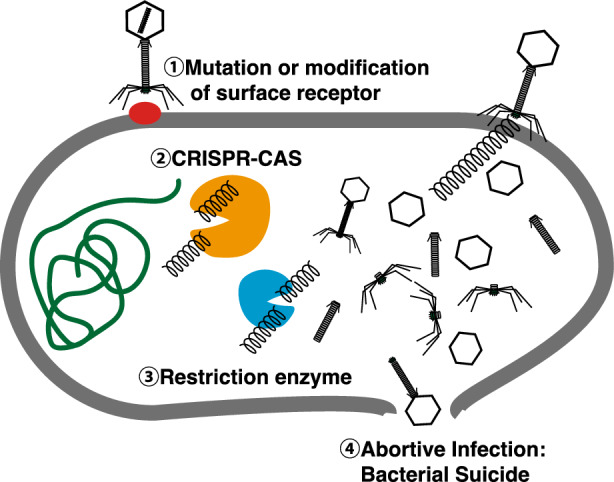
Bacteria acquire phage resistance. There are several mechanisms through which bacteria can gain resistance to phages, as follows: ① mutation or modification of surface receptors, ② the CRISPR‒Cas system, ③ restriction-modification system, and ④ abortive infection

Even phages that initially demonstrate effectiveness against specific bacterial species can become ineffective if the bacteria develop resistance. There are several mechanisms through which bacteria can gain resistance to phages, and the primary mechanisms are outlined below [15, 16] (Fig. 1):

- i) Mutation or modification of surface receptors: phages attach to bacteria by binding to specific receptors on the bacterial cell wall. Bacteria can alter the structure or quantity of these receptors to prevent phage attachment and block subsequent infection.
- ii) CRISPR‒Cas system: bacteria have evolved a defense system known as CRISPR/Cas to restrict the invasion of foreign DNA, such as that from bacteriophages or plasmids [17]. This system primarily consists of two main components: (i) the CRISPR locus (clustered regularly interspaced short palindromic regions) and (ii) cas genes, which encode CRISPR-associated (Cas) proteins. The mechanism of CRISPR/Cas activity involves the active integration of small fragments (proto-spacers) of invading DNA (from phages or plasmids) into the genome, which is then transcribed into short RNAs that direct the degradation of foreign invading DNA elements. In this way, bacteria store fragments of phage DNA from past encounters in their CRISPR sequences, enabling them to “remember” and rapidly eliminate those phages should they attack again.
- iii) Restriction-modification system: this system employs restriction enzymes to cut the DNA of invading phages. While bacterial DNA is protected from these enzymes by methylation, unprotected phage DNA is easily targeted and cut.
- iv) Abortive infection: in this defensive strategy, a bacterium infected by a phage self-destructs to halt the proliferation of the phage and prevent the spread of infection to other bacteria, effectively sacrificing the infected cell to protect the colony.

These adaptive mechanisms enable bacteria to counter various phage attacks, leading to an ongoing evolutionary arms race between bacteria and phages. The effectiveness of each strategy can vary on the basis of the environmental conditions and the specific type of phage involved. To combat phage resistance, strategies involving the use of phage cocktails—mixtures of phages—have been developed. These cocktails are designed to maintain effectiveness against multiple bacterial strains and to hinder the development of resistance.

### Immunogenicity of phages

Phages encode dozens of proteins with unknown functions, some of which could be potentially harmful. The immunogenicity of these phage proteins, especially when the phages are repeatedly administered, raises concerns. Recent case studies have underscored this issue; for example, in a study of the treatment of lung infections caused by *Mycobacterium abscessus* in immunocompetent patients, a cocktail of three phages was administered intravenously for 6 months [18]. One month after therapy, a significant neutralizing antibody response involving IgM and IgG was observed, which diminished the effectiveness of phage therapy after 2 months. The impact of these antibodies on the efficacy and clinical success of phage therapy largely depends on the administration route, with localized or gastrointestinal applications resulting in minimal adverse effects.

Moreover, in acute infections, the generation of phage-specific antibodies is less critical since antibodies develop after phages have achieved their antibacterial effects. However, in long-term phage therapy for chronic infections or when the same phages are used repeatedly, phage-specific antibodies play a crucial role by enhancing the humoral immune response [11]. Consequently, there are ongoing efforts to engineer phages that improve therapeutic properties, safety, and adaptability to different host domains.

### Inflammation caused by phage-induced bacterial lysis

Another challenge in phage therapy is the dispersion of bacterial components by phage-induced lysis, which act as pathogen-associated molecular patterns (PAMPs), potentially triggering acute inflammatory responses and other biological reactions [11]. This complication, similar to the post-antibacterial treatment effects observed with monobactam antibiotics, raises issues related to the bactericidal action of antimicrobial agents. Adjuvant therapies, such as the adsorption and removal of endotoxins released during lysis, are being explored as potential solutions to mitigate these effects.

## Clinical applications of phage therapy

In phage therapy, practitioners utilize two types of phages: naturally occurring phages and artificially modified phages. Numerous ventures have been established worldwide to advance phage development. These companies are actively engaged in projects aimed at developing engineered phages that exhibit a broader spectrum of activity against diverse bacterial strains, remove superfluous phage proteins that may pose safety concerns, and increase the stability of their bactericidal properties. These engineered phages are not only in development, but are also actively being employed as therapeutic products. The following is an overview of the advancements achieved thus far in this dynamic field.

### Personalized phage therapy

Numerous case reports or case series reports, including the well-known Patterson case [5], attest to the efficacy of personalized phage therapy (Table 2). Belgium has established a national phage bank specifically designed to support personalized phage treatments. In this significant context, the University of California, San Diego (UCSD) played a pivotal role in the establishment of IPATH, heralding the start of widespread clinical use of phage therapy in the United States. In recent years, there has been a surge in successful outcomes from such personalized approaches. For example, a 2022 case in France involved a patient with a spinal abscess caused by panresistant *P. aeruginosa*, which was resistant to commercially available phages [19]. A tailored phage cocktail was formulated and administered in collaboration with European academic institutions. Despite undergoing two surgeries, the patient, who continued to carry small-colony variant bacteria, was successfully treated with adjuvant therapy involving locally and intravenously injected purified phages. This case highlights the potential and adaptability of personalized phage therapy in treating complex, resistant infections.

**Table 2:** Recent clinical case reports of personalized phage therapy

Organization/company	Country	Study	Year	Refs.
Baylor college of medicine (TAILΦR)	USA	A retrospective, observational study. Device-related or systemic infections, 12 patients • Evaluated 12 cases of customized phage therapy, showing a 66% favorable response rate, with 42% bacterial eradication • Phage therapy was safe, though immunological neutralization occurred in some cases	2023	[20]
Belgian Consortium Study	Belgium	A multicenter, multinational, retrospective observational study. Individualized phage therapy • Analyzed 100 cases of individualized phage therapy across 12 countries • Showed clinical improvement in 77.2% of cases and bacterial eradication in 61.3% • The use of antibiotics alongside phage therapy increased the likelihood of success	2024	[21]
Prosthetic joint infections	Italy	A case report. Pa53 (anti-*P. aeruginosa* phage) • A 62-year-old patient with chronic *P. aeruginosa* infection was successfully treated with customized phage therapy and meropenem, showing no infection recurrence over 2 years	2023	[22]
Diabetic foot infection	UK	A case series. Anti-*S. aureus* therapy • Tested anti-*S. aureus* phage therapy on 10 patients at high risk of amputation • Nine out of 10 patients benefited, though one patient showed no response to treatment	2023	[23]
LVAD infection study	Israel/USA	A case series. Anti-*P. aeruginosa* therapy • Phage therapy in combination with antibiotics for LVAD-related *P. aeruginosa* infections had limited success, with breakthrough bacteremia and immune reactions hindering effectiveness	2023	[24]

In 2023, researchers at the Baylor College of Medicine's Tailored Antibacterials and Innovative Laboratories for Phage (TAILΦR) conducted evaluations of 12 cases of customized phage therapy from their production center [14, 20]. This project involved meticulous screening, purification, sequencing, and assessment of phages adhering to stringent standards. The phages received FDA approval for compassionate use under an Investigational New Drug application. Out of 50 requests for phage therapy, customized phages were produced for 12 patients, primarily targeting device-related or systemic infections. Among the 50 requests, the detailed reasons for the 38 that were not adapted were as follows: improvement in condition in 8 cases, treatment initiation delayed by more than 10 weeks in 8 cases, patient death before treatment initiation in 8 cases, just before treatment initiation in 5 cases, inability to isolate phages in 5 cases, bacteria not identified in 4 cases, and other reasons in 3 cases. The collected data covered aspects such as time to treatment, synergistic effects with antibiotics, and patient immune responses. Among the patients, bacteria were eradicated in 5 (42%), whereas clinical improvement was noted in 7 (58%). Overall, two-thirds (66%) of the patients demonstrated favorable outcomes, with no significant side effects reported. However, immunological neutralization of the phages occurred in 5 patients, and some complications arose from secondary infections. This study verified that the production and administration of customized phages are safe and can often produce clinically or microbiologically favorable results. These findings suggest that phage therapy could be an effective treatment option for specific infections.

A study by a Belgian consortium conducted a retrospective observational analysis of the first 100 consecutive cases of individualized phage therapy from January 2008 to April 2022, spanning 12 countries, 35 hospitals, and 29 cities [21]. This study focused on particularly challenging infections, including infections of the lower respiratory tract, skin, and soft tissues, as well as bone infections. A total of 26 individual bacterial phages and six defined bacterial phage cocktails were selectively employed. The treatment yielded clinical improvement in 77.2% of the patients and eradicated target bacteria in 61.3% of the patients. Analysis revealed that the likelihood of eradication was 70% lower when no antibiotics were used concurrently (odds ratio = 0.3; 95% confidence interval = 0.127–0.749). This study documented both the emergence of bacteriophage resistance in vivo and the synergistic effects of phages and antibiotics in vitro. Bacteriophage resistance was observed in 43.8% of patients, whereas synergistic effects with antibiotics were confirmed in 90% of patients. Furthermore, the study revealed resensitization to antibiotics and a reduction in toxicity in bacterial strains resistant to bacteriophages during phage therapy. Bacteriophage immune neutralization occurred in 38.5% of the patients screened. The study reported 15 adverse events, seven of which were nonserious adverse drug reactions potentially related to phage therapy. These findings underscore that combining antibiotics with bacteriophage therapy can increase the effectiveness of phage therapy, thereby highlighting its potential as a treatment option for managing complex infections.

Prosthetic joint infections (PJIs) caused by *P. aeruginosa* represent a significant challenge in orthopedic surgery. Italian researchers reported a case involving a 62-year-old female patient who suffered from a chronic infection in her replaced hip due to *P. aeruginosa* [22]. She was treated with customized phage therapy in conjunction with the antibiotic meropenem. The specific phage used, Pa53, effectively eradicated the infection. Following treatment, the patient was monitored over a 2-year clinical follow-up period, during which no serious side effects or signs of infection recurrence were observed, confirming the successful elimination of *P. aeruginosa* infection. This case underscores the safety and efficacy of combining phage Pa53 with meropenem as a treatment strategy for PJI caused by *P. aeruginosa*, highlighting the potential of phage therapy as a viable alternative for managing antibiotic-resistant pathogens.

A study in the UK tested topical adjunctive anti-*S. aureus* phage therapy in ten DFI patients at high risk of amputation [23]. The therapy included phages sourced from the Eliava Institute in Tbilisi, Georgia, and manufactured at the Queen Astrid Military Hospital in Brussels, Belgium, where phage production is regulated and approved by the Belgian Federal Agency for Medicines and Health Products. In two UK hospitals, this clinical application resulted in nine out of the ten patients benefitting from adjunctive phage therapy, with no adverse effects reported by clinicians or patients. Specifically, six patients experienced resolution of infection and limb salvage, and another patient experienced resolution of soft tissue infection, although they required amputation due to unresolved osteomyelitis. The eighth patient achieved *S. aureus* eradication in a polymicrobial infection. However, the ninth patient exhibited clinical improvement before phage therapy was prematurely terminated because of an unrelated event. One patient, infected with a weakly susceptible *S. aureus* strain, did not respond significantly to the treatment. This study suggests that while anti-*S. aureus* phage therapy can be effective for some patients with DFIs, its efficacy is not universal across all patients.

Recent advances in phage therapy have provided considerable optimism, yet some reports have shown limited effectiveness. Left ventricular assist devices (LVADs), which are commonly used in heart failure treatment, increase susceptibility to infections, often complicating patient outcomes. Phage therapy has been investigated as an alternative treatment for antibiotic-resistant infections, particularly endovascular infections caused by *P. aeruginosa* in LVAD patients [24]. In a recent study, intravenous phage therapy, in conjunction with antibiotics, was administered to four patients across five treatment courses. The regimen included one-to-four different wild-type virulent phages, which were applied over periods ranging from 14 to 51 days. Unfortunately, the anticipated success of these treatments was not realized. Notably, breakthrough bacteremia occurred in four of the five treatments, raising significant safety concerns. Additionally, two patients succumbed to their underlying infections, thereby calling the clinical efficacy of phage therapy into question. Variability in phage susceptibility was noted during three treatment sessions, and serum-neutralizing reactions were observed in all tests, suggesting that the patients' immune responses may have hindered the effectiveness of the phages. Moreover, the detection of prophages in isolates from two patients highlighted the genetic complexity of *P. aeruginosa*, further complicating treatment efforts. These findings underscore the need for substantial safety measures when employing phage therapy in LVAD infections and the requirement for more comprehensive studies to thoroughly evaluate the safety and efficacy of this therapy.

### Bacteriophage products developed for a wide target range

Numerous clinical trials, including those currently ongoing, are being conducted in association with venture companies (Table 3).

**Table 3:** Recent clinical trials of phage therapy by venture companies

Organization/company	Country	Study		Year	Refs.
Pherecydes Pharma (Phaxiam Therapeutics)	France	A randomized, controlled, double-blind phase 1/2 trial • PhagoBurn *E.coli*, and *P aeruginosa* infections	NCT02116010	2019	[25, 26]
Armata Pharmaceuticals	USA	A multi-center, double-blind, randomized, placebo-controlled, single and multiple ascending dose phase 1/ 2 trial • AP-PA02, *P. aeruginosa* for severe respiratory infections • Evaluating safety and tolerability of inhaled AP-PA02 in chronic lung infections and cystic fibrosis (SWARM-Pa)	NCT04596319	2024–	[27]
Adaptive phage therapeutics	USA	A randomized, parallel, double-blind, placebo-controlled, repeat dose, multi-site phase 1/2 trial • DANCE™; diabetic foot osteomyelitis	NCT05177107	2022–	[28]
Israeli phage therapy center	Israel	A pre-phase-1 cohorts trial • PASA16;*P. aeruginosa* infection	–	2023	[29]
TechnoPhage	Portugal	A randomized, parallel, open label, phase 1/2a trial • TP-122A, ventilator-associated pneumonia • Assess safety and tolerability	NCT06370598	2024–	[30]
MB pharma	Czech Republic	A randomized, double-blind, placebo-controlled phase 1/2a trial • DUOFAG®; phage cocktail against *S. aureus* and *P. aeruginosa* for* s*urgical wound infection	NCT06319235	2022–	[31]
Locus biosciences	USA	A double-blind, randomized, active-controlled phase 2/3 trial • LBP-EC01; *E. coli*-induced UTIs • Treatment of acute uncomplicated UTI caused by drug-resistant *E. coli* (ELIMINATE Trial)	NCT05488340	2024	[32]

The PhagoBurn project, developed by Pherecydes Pharma (now known as Phaxiam Therapeutics) and Erytech Pharma, has participated in EU-led clinical trials across nine burn centers in France and Belgium [25, 26]. This trial was a randomized phase 1/2 study that aimed to compare the effectiveness and tolerability of a natural lytic anti-*P. aeruginosa* bacteriophage (PP1131) in treating wound infections in burn patients. After 7 days of daily topical application, followed by a 14-day follow-up, it was observed that PP1131, which was used at very low concentrations, reduced the bacterial load in burn wounds more slowly than did the standard treatment. Consequently, the trial was terminated because of the insufficient efficacy of PP1131. On the basis of these findings, the research team determined that an increase in the PP1131 concentration is necessary, alongside further studies to optimize its concentration.

Armata Pharmaceuticals, Inc. is developing a therapeutic phage cocktail, AP-PA02, targeted explicitly at *P. aeruginosa* [27]. This phage cocktail is used to treat severe respiratory infections in patients with cystic fibrosis and noncystic fibrosis bronchiectasis. In 2020, Armata received FDA approval for its Investigational New Drug application for the “SWARM-P.a.” study. This is a phase 1b/2a clinical trial designed to evaluate the safety, tolerability, and phage recovery profile of AP-PA02 administered by inhalation to subjects with cystic fibrosis and chronic pulmonary *P. aeruginosa* infection. The primary endpoints included the incidence and severity of treatment-emergent adverse events. The secondary endpoints included changes in the colony-forming units of *P. aeruginosa*. In this study, a sustained reduction in bacterial load was observed, and pharmacokinetic data revealed that AP-PA02 effectively delivers treatment to the lungs of patients while minimizing the exposure of other organs. On the basis of these positive results, Armata Pharmaceuticals has decided to further advance AP-PA02 development and is preparing for a phase 2b trial. The next phase will focus on a more detailed evaluation of the long-term efficacy and safety of the treatment, potentially establishing AP-PA02 as a practical option for treating severe respiratory infections.

Adaptive Phage Therapeutics, Inc. (APT) is currently evaluating a treatment in a phase 1/2 clinical trial, the “DFO Adaptive Novel Care Evaluation (DANCE™)”, which aims to evaluate the safety and efficacy of phage therapy for patients with diabetic foot osteomyelitis [28]. This trial employs phages selected from APT's extensive phage bank. In collaboration with the Mayo Clinic, the trial is utilizing a unique phage susceptibility test to precisely match each patient’s infection with the optimal phage, ensuring targeted treatment. Moreover, APT’s recent acquisition by BiomX of Israel promises to enhance phage therapy development and commercialization. This strategic integration is poised to broaden APT’s technological and resource base, significantly accelerating the development of therapies not only for diabetic foot osteomyelitis, but also for a range of other chronic bacterial infections.

The Israeli Phage Therapy Center, managed by Hadassah Medical Center and Hebrew University, has documented a case series involving the compassionate use of the PASA16 phage in 16 patients with challenging-to-treat *P. aeruginosa* infections [29]. This series details the clinical microbiological susceptibility of the PASA16 phage, administration protocols, clinical data, and patient outcomes. Treatment was administered intravenously, topically at infection sites. The study included analysis of data from 15 of the 16 participants, with 13 patients (86.6%) experiencing favorable clinical outcomes. Notably, the combination of the PASA16 phage and antibiotics was successful, especially in patients who had not responded to conventional antibiotic treatments.

TechnoPhage, which is based in Lisbon, Portugal, is developing a novel drug called TP-122A, which comprises three bacteriophages that specifically target *P. aeruginosa* for the treatment of ventilator-associated pneumonia [30]. The drug is administered in a nebulized form, ensuring direct delivery to the lungs. Currently, TP-122A is being evaluated in a phase 1/2a clinical trial involving 15 adult patients to assess its safety and tolerability.

MB Pharma has developed a specialized phage cocktail, “DUOFAG^®^”, which is known for its efficacy against *S. aureus* and *P. aeruginosa* [31]. Designed primarily to treat DFIs, DUOFAG^®^ has potential applications for a broader range of bacterial infections. The DUOFAG^®^ formulation includes two phages that target *S. aureus* and one that targets *P. aeruginosa*. These phages have undergone extensive morphological and genomic characterization, with demonstration of their effectiveness across numerous clinical isolates. Produced in good manufacturing practice (GMP)-certified facility in the Czech Republic, DUOFAG^®^ meets high standards of quality and safety.

Locus Biosciences is advancing the development of phage-based therapeutics through the utilization of CRISPR–Cas3 technology, which allows phages to irreversibly destroy bacterial DNA. The company has produced a phage cocktail that targets a variety of pathogens and has specifically developed LBP-EC01 for treating urinary tract infections (UTIs) caused by antibiotic-resistant *Escherichia coli.* In July 2022, Locus Biosciences launched a phase 2/3 trial, the ELIMINATE trial, to assess the safety, tolerability, pharmacokinetics, and efficacy of this therapeutic [32]. LBP-EC01 is a genetically enhanced cocktail composed of six bacteriophages. The trial is structured in two parts: the first part will determine the optimal dosage for uncomplicated UTIs, whereas the second part will be conducted as a randomized, controlled, double-blind study. Preliminary results revealed that urinary *E. coli* levels significantly decreased within four hours post-administration, and by day ten, UTI symptoms had completely resolved in all patients evaluated. These promising outcomes suggest that LBP-EC01 may be an effective treatment option for UTIs.

## Expectations and challenges

As mentioned earlier, in recent years, there has been an increase in the number of detailed case reports on phage therapy. This is partly due to the growing number of countries creating a “parallel track” that allows for the compassionate, case-by-case use of phage therapy even without efficacy data from clinical trials, especially when antibiotic options have failed. The number of phase 1/2 clinical trials involving phages registered on ClinicalTrials.gov is increasing. Phage banks established in the USA and Belgium are becoming essential as libraries that supply therapeutic phages for clinical use and enable the rapid provision of customized polyvalent phage cocktails. While fixed phage cocktails developed by venture companies are easy to manufacture and deploy, they sometimes lack polyvalency; however, advances in phage engineering aimed at developing phages with a broader host range are highly anticipated. These expectations present significant unresolved challenges.

Concerns about intravenous administration are related to the potential for an adaptive immune response to administered phages, which could impair therapeutic effects, especially in patients with immune capability and those requiring longer treatment periods. Intravenous, topical, and inhalation applications have been reported, but pharmacodynamic studies for infections at hard-to-access sites, such as artificial joints or urinary tract infections may require more innovative approaches. In combination with antimicrobials, the interesting phenomenon of evolutionary trade-offs, where the acquisition of phage resistance in multidrug-resistant host bacteria can increase antibiotic susceptibility in parallel [33, 34], suggests that establishing methods to monitor the emergence of phage-resistant bacteria and changes in drug susceptibility could be helpful. In addition to the numerous reports on combination therapy with phage therapy and conventional antibiotics, we have recently reported the efficacy of combining antibody therapy, which exerts antibacterial toxin inhibitory effects, with phage therapy [35]. Although it is unlikely that phages will completely replace antibiotics, the technological advances in phage therapy outlined in this article have the potential to significantly improve antimicrobial stewardship, making their importance increasingly paramount in the future.

## Summary

Phage therapy is a treatment modality that, unlike antibiotics, targets and is effective against specific bacteria. The formulations of this therapy primarily include lytic phages, which infect bacterial cells and cause them to burst, thereby killing the bacteria. A characteristic of phage therapy is that bacteria may acquire resistance to phages, and the immunogenicity of phages can sometimes pose problems. Bacteria possess a variety of defense mechanisms to escape phage attack and can develop phage resistance. Moreover, since phages are effective against only specific bacterial strains, it is necessary to customize and select effective phages for each bacterial strain involved in an infection. Naturally derived and artificially modified phages are used in phage therapy, and many venture companies are engaged in their development. In particular, personalized treatment, in which phages are selected and used according to the specific patient's condition, is emphasized. In Belgium, progress has been made in establishing a national phage bank. IPATH was established at UCSD in the USA, advancing clinical applications. Furthermore, the development of customized phage cocktails for antibiotic-resistant bacteria and personalized treatments for severe infections have been reported. Many venture companies are working to commercialize phage therapy, and clinical data demonstrate the safety and efficacy of phage therapy, suggesting that combining phage therapy with antibiotic treatment could enhance its effects. In critical care medicine, where frontline treatment for severe infections is crucial, increasing attention should be given to trends in clinical trials of phage therapy.

## Data Availability

Not applicable.

## Abbreviations

IPATH: Innovative phage applications and therapeutics
UCSD: University of California, San Diego
PJI: Prosthetic joint infection
DFI: Diabetic foot infection
LVADs: Left ventricular assist devices
APT: Adaptive phage therapeutics, Inc.
UTI: Urinary tract infections
